# Restorative Justice by Proxy: A Forensic Psychiatry Case Study

**DOI:** 10.1192/bjo.2024.660

**Published:** 2024-08-01

**Authors:** Suhanthini Farrell

**Affiliations:** Greater Manchester Mental Health NHS Foundation Trust, Manchester, United Kingdom

## Abstract

**Aims:**

Restorative justice (RJ) is the process of bringing together offender and victim in the aftermath of an offence. Handled correctly, there can be substantial benefits for both parties. Communication is typically direct with an RJ facilitator from the criminal justice system, but the Crown Prosecution Service makes reference to the option of “shuttle mediation”, with the RJ facilitator relaying information indirectly between parties.

The use of RJ in forensic psychiatry has been limited, with reasons including concern for the patient offender's vulnerability, impact on their mental state from the stress of facing their victim, and unpredictability of response. Adaptation of the “shuttle mediation” model with use of a trusted mental health professional may help to overcome many such obstacles.

**Methods:**

Patient A committed his index offence of murder at age 22. During his mandatory life sentence, he developed schizophrenia and was transferred to a secure hospital. His illness proved treatment resistant and many years passed before he was discharged to the community on life licence following Parole Board release. Although generally stable, he was easily overwhelmed by stressful situations, in which he could struggle to articulate his thoughts.

His victim’s wife and daughter enquired about the possibility of restorative justice. A's probation officer liaised with A's consultant psychiatrist. A was considered unsuitable for direct approach, but following discussion with an RJ facilitator, a proxy model was agreed upon. This was based on shuttle mediation but with the consultant psychiatrist acting as shuttle, or proxy, for A.

The aims of the family were elicited in advance. Capacitous consent to information sharing was obtained from A, with boundaries of what would be shared clearly agreed. Information A wished to share with the family was established, including verbatim quotes.

**Results:**

Following the RJ meeting, feedback from the victim's family and A was strongly positive. The victim's family reported a sense of peace and closure from the experience. A's longstanding anxiety, with feelings of guilt and self-blame reduced. Both parties expressed gratitude for the meeting and did not find the use of a proxy negated the benefits of the experience.

**Conclusion:**

This case report demonstrates that a proxy model of restorative justice is feasible and can be successful for patient offenders with severe and enduring mental disorder. The potential benefits to patient and victim suggest that it could be considered more widely as an option in forensic psychiatry settings.

